# Concurrent ascending colon adenocarcinoma and ileocecal tuberculosis: A case report

**DOI:** 10.1097/MD.0000000000029430

**Published:** 2022-05-27

**Authors:** Sun Min Park, Ji Hoon Kim, Yosep Chong, Won-Kyung Kang

**Affiliations:** a Department of Surgery, Yeouido St. Mary's Hospital, College of Medicine, The Catholic University of Korea, Seoul, Republic of Korea; b Department of Surgery, Incheon St. Mary's Hospital, College of Medicine, The Catholic University of Korea, Incheon, Republic of Korea; c Department of Pathology, Yeouido St. Mary's Hospital, College of Medicine, The Catholic University of Korea, Seoul, Republic of Korea.

**Keywords:** ascending colon cancer, ileocecal tuberculosis

## Abstract

**Rationale:**

Few cases have been reported of the coexistence of tuberculosis and adenocarcinoma of the large bowel. We report a rare case of concurrent ascending colon adenocarcinoma and ileocecal tuberculosis, which were nearly indistinguishable from one another.

**Patient concerns:**

A 59-year-old man visited our clinic with dizziness and anorexia.

**Diagnosis:**

Computed tomography revealed a mass in the ascending colon with ill-defined nodules in the liver. A colon biopsy showed adenocarcinoma with multinucleated giant cells. The liver nodules were confirmed to be metastatic adenocarcinomas.

**Interventions:**

Ant tuberculosis medications were administered prior to surgery. Two weeks later, a laparoscopic right hemicolectomy and radiofrequency ablation of the liver were performed.

**Outcomes:**

The final pathology confirmed adenocarcinoma with chronic granulomatous inflammation and giant cells.

**Lessons:**

In this patient, the cancer was in an advanced stage and had no history of tuberculosis infection. Thus, in this case, the malignancy seemed to create the proper environment for either reactivation of a latent tuberculosis infection or, less likely, for the acquisition of a primary mycobacterial infection. In conclusion, clinicians should be aware of the possibility of concurrent colon adenocarcinoma and intestinal tuberculosis.

## 1 Introduction

Intestinal tuberculosis accounts for 2% of tuberculosis (TB) cases worldwide.^[1]^ Although this condition is uncommon, it remains life-threatening if undiagnosed. The ileocecal region is the most common site of intestinal tuberculosis. Due to its nonspecific symptoms, intestinal tuberculosis is difficult to discriminate from other intestinal diseases. Furthermore, the clinical diagnosis of coexisting intestinal tuberculosis and colon cancer is more difficult because of the overlapping clinical features and findings.

Few cases have been reported regarding the coexistence of tuberculosis and adenocarcinoma of the large bowel. Several theories have been proposed for its pathogenesis.^[2]^ Hence, we report a rare case of concurrent ascending colon adenocarcinoma and ileocecal tuberculosis, which are nearly indistinguishable from one another.

## 2 Case report

**Figure 1:**
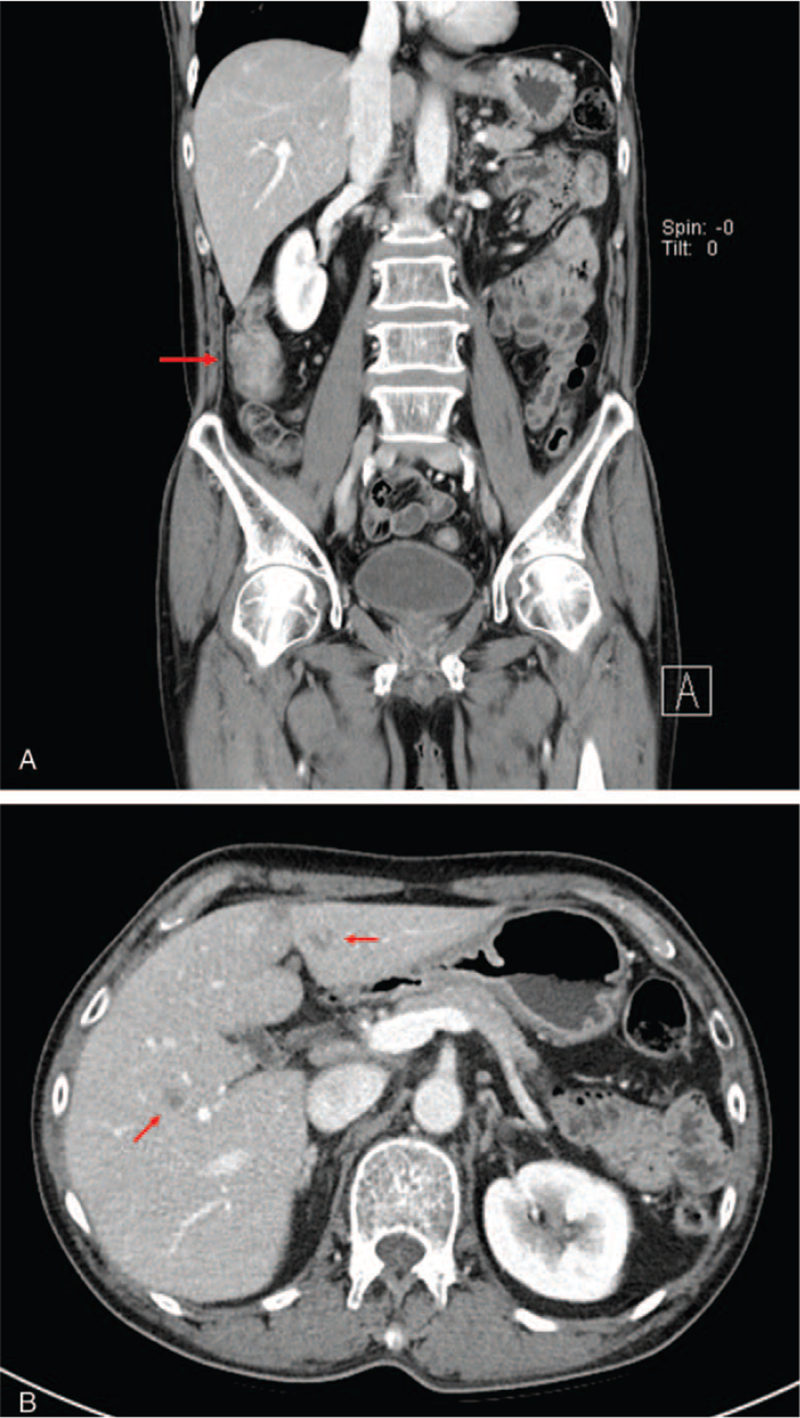
CT findings. (A) 4.3 cm size colonic mass in ascending colon (red arrow). (B) Ill-defined hypodense nodules in liver S3 (1.0 cm) and S5 (1.3 cm) (red arrows). CT = computed tomography.

**Figure 2:**
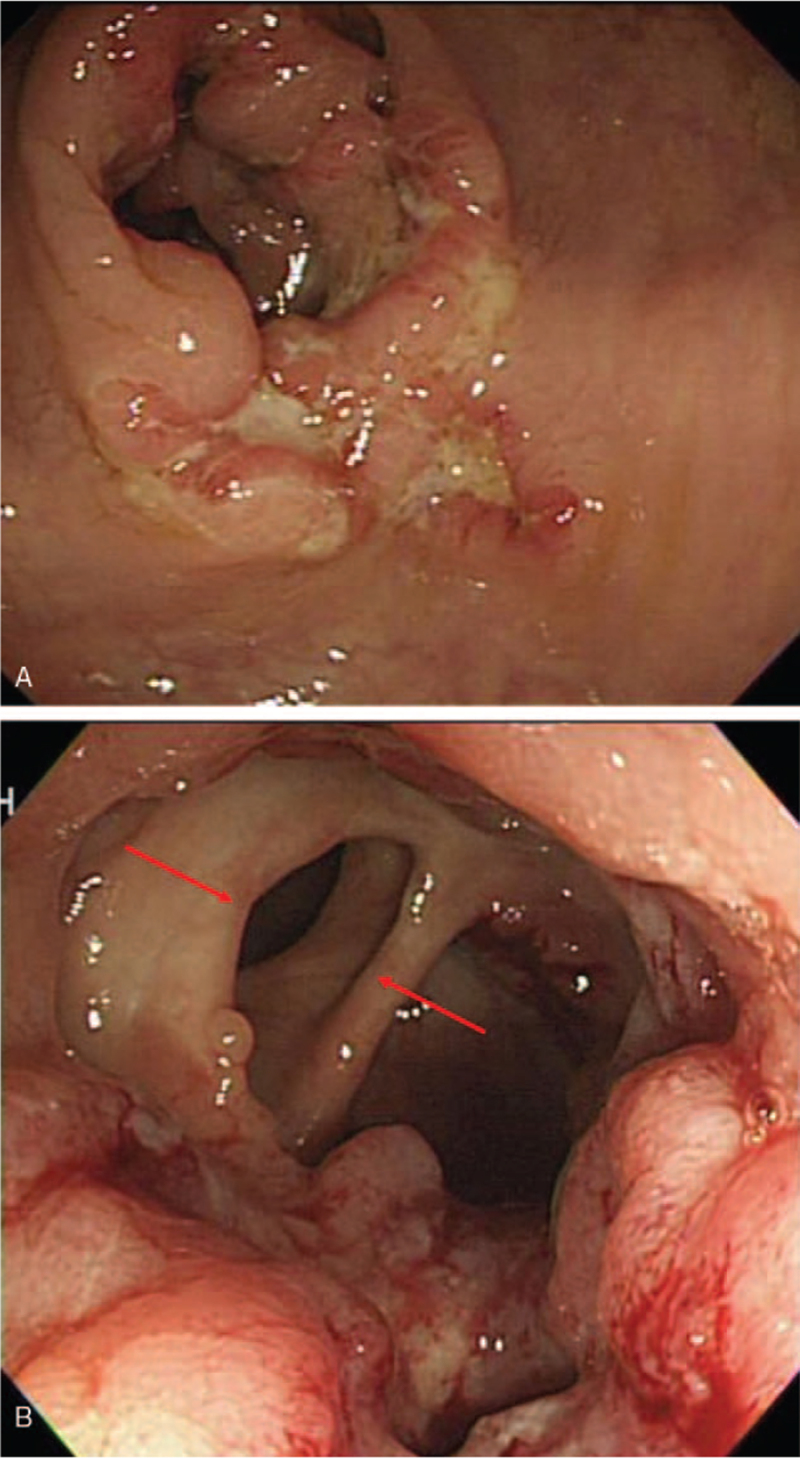
Colonoscopic findings. (A) Ulcerofungating mass and multiple ulcer with exudate at proximal ascending. (B) Thickening of the lips of the ileocecal valve and wide gaping of the valve.

**Figure 3:**
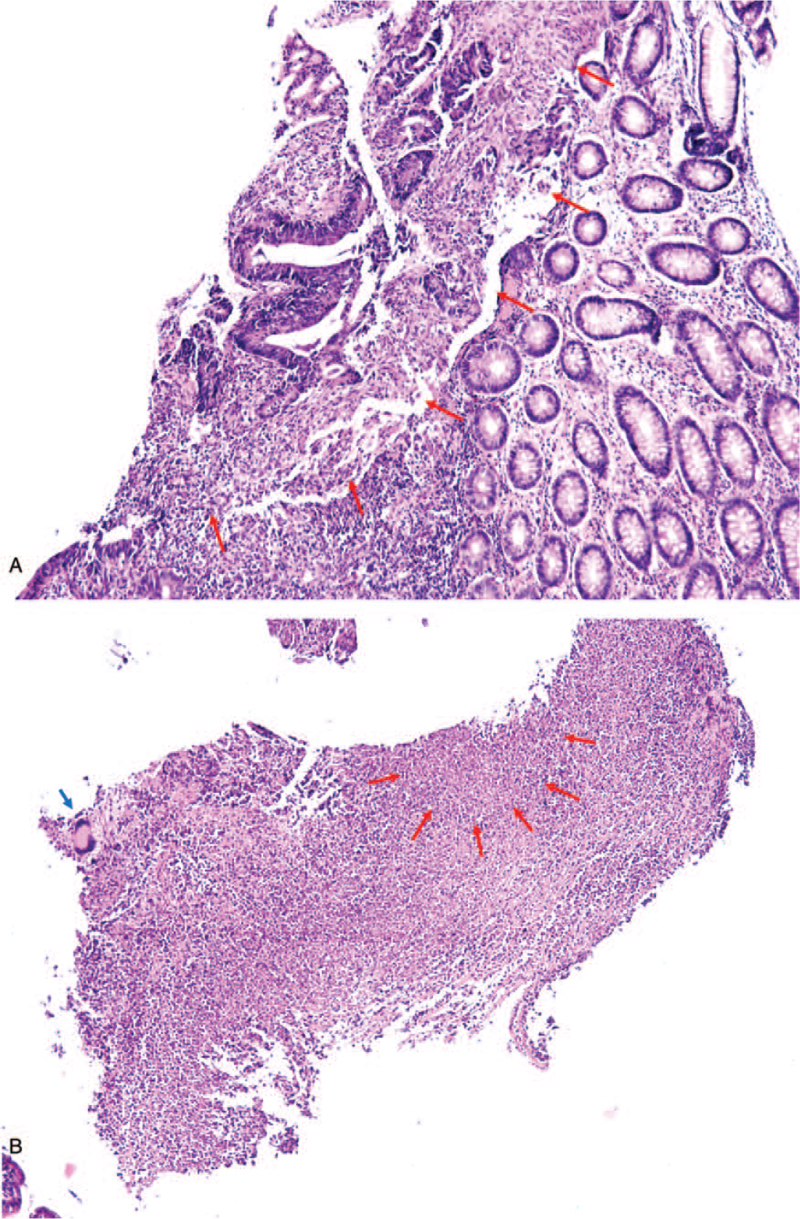
Histological findings of ascending colon biopsy. (A) Adenocarcinoma (red arrows). (B) Chronic granulomatous inflammation with caseous necrosis (red arrows) and multinucleated giant cell (blue arrow) (H&E, ×12.5).

A 59-year-old man presented to the outpatient clinic with dizziness and anorexia. The patient's blood pressure was 110/70 mm Hg, heart rate was 80 beats/min, respiratory rate of 20/minute, and body temperature was 37.0°C. Blood chemistry showed elevation of liver enzymes (aspartate transaminase/alanine transferase 163/91 U/L) and carcinoembryonic antigen (24.4 ng/mL). The patient had no history of liver disease or tuberculosis. The chest radiograph was normal. Ultrasonography of the liver showed a 1.2 cm lobulated hypoechoic lesion in segment 3. Computed tomography (CT) showed about a 4.3 cm colonic mass in the ascending colon with ill-defined, hypodense nodules in the liver S3 (1.0 cm) and S5 (1.3 cm) (Fig. 1A and B). Colonoscopy showed a 3 cm-sized ulcerofungating mass and multiple ulcers with exudate at the proximal ascending colon and thickening of the lips of the ileocecal valve with a wide gaping of the valve (Fig. 2A and B). Biopsy showed adenocarcinoma with chronic granulomatous inflammation and multinucleated giant cells (Fig. 3A and B), and TB polymerase chain reaction was positive; liver nodules were confirmed to be metastatic adenocarcinomas via sonography-guided liver biopsy.

**Figure 4:**
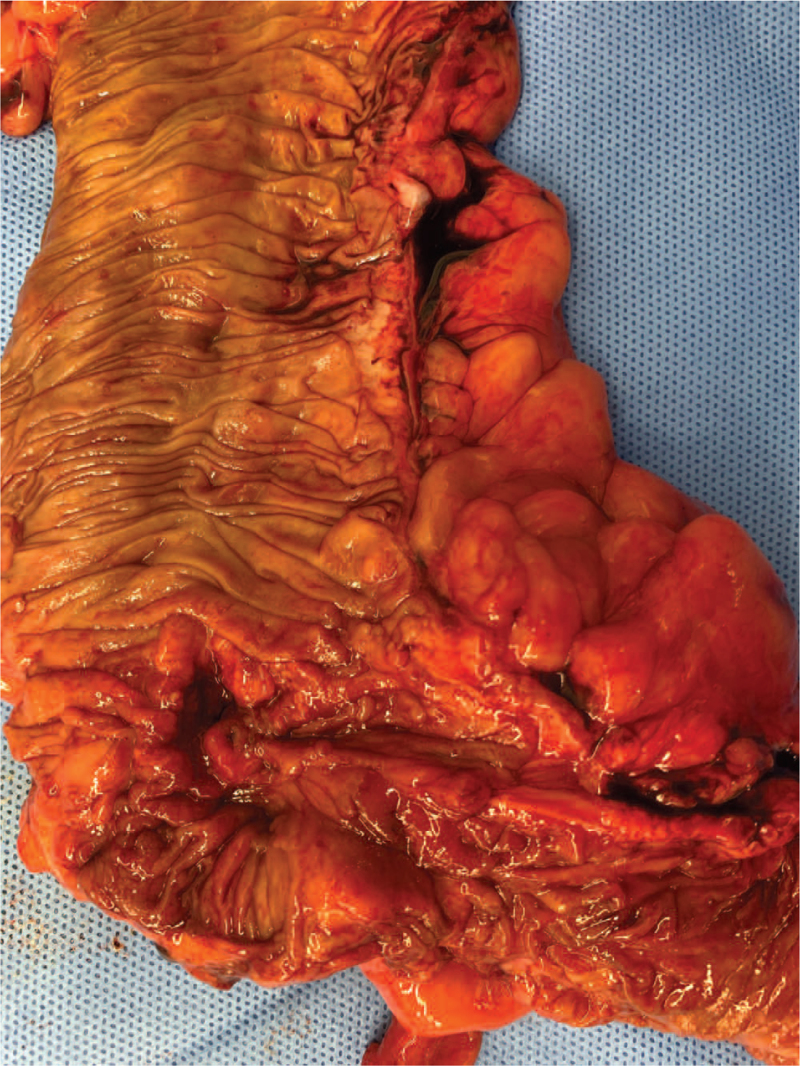
Surgical specimen of ascending colon and terminal ileum.

**Figure 5:**
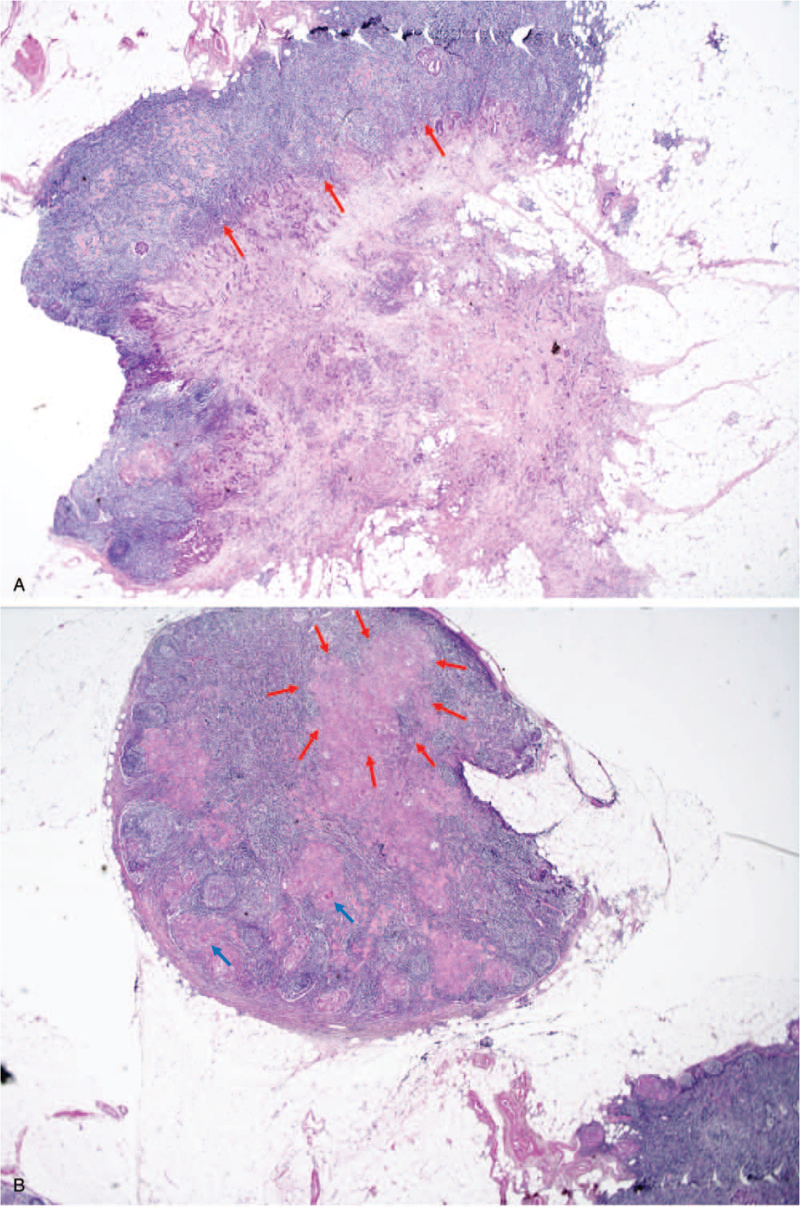
Histological findings of regional lymph node. (A) Adenocarcinoma (red arrows). (B) Chronic granulomatous inflammation (red arrows) with giant cells (blue arrows), much regressed d/t medication (H&E, ×12.5).

Four drug regimens of anti-TB medications (isoniazid, ethambutol, pyrazinamide, and rifampicin) were administered prior to surgery. Two weeks later, laparoscopic right hemicolectomy and radiofrequency ablation of liver metastatic lesions were performed. Surgical specimens demonstrated an ulcerofungating mass with scar changes, but the previously mentioned ulcerative lesion resolved due to anti-TB medication (Fig. 4). As the final pathology, moderately differentiated adenocarcinoma was confirmed in the colon specimen, but there were no multinucleated giant cells in the colon due to the anti-TB medication. In the regional lymph nodes, both cancer cells (4/22) and chronic granulomatous inflammation with giant cells (9/22) were observed (Fig. 5A and B). TB polymerase chain reaction was negative.

At present, the patient is being treated with infusional 5-FU/leucovorin plus oxaliplatin (FOLFOX) with avastin chemotherapy and anti-TB medication and is well tolerated without any adverse event.

## 3 Discussion

Intestinal TB is a rare disease that may be misdiagnosed as other diseases, such as Crohn disease, ulcerative colitis, and colon cancer. Clinical diagnosis of coexisting intestinal tuberculosis and colon cancer is more difficult because of overlapping clinical features and findings.

CT is an effective tool for diagnosing intestinal tuberculosis. The most common findings on CT that are consistent with intestinal TB are lymphadenopathy, bowel wall thickening, high-density ascites, and irregular soft tissue densities in the omental area. However, lymphadenopathy and bowel wall thickening are also common findings in colon cancer. These findings can be nonspecific and may mimic many other conditions, including inflammatory bowel disease or colon cancer.^[3,4]^ Colonoscopy is also a useful diagnostic tool. Colonoscopy revealed ileocecal inflammation, thickening, and wide gaping of the valve. These findings may be useful for differential diagnoses.

There is a lack of data in the literature regarding the optimal duration of anti-TB medication use before colon cancer surgery. In the case of the coexistence of pulmonary TB and lung cancer, as there is clinical evidence of rapid clearance of viable TB bacilli in sputum after the initial 2 weeks of an intensive 4-drug regimen of anti-TB treatment in drug-susceptible TB, it is generally safe to perform lung resection with sputum AFB smear conversion after 2 to 3 weeks of anti-TB treatment.^[5]^ In this patient, 4-drug regimen of anti-TB medications was administered prior to surgery. Two weeks later, laparoscopic right hemicolectomy and radiofrequency ablation of liver metastatic lesions were performed.

The causal link between TB and lung cancer is well documented. TB has been extensively studied as a risk factor for lung cancer.^[6]^ Liang et al^[7]^ performed a systematic review of 37 case-controlled and four cohort studies (19,143 cases and 118,191 controls) and reported significant lung cancer risk with preexisting TB (RR = 1.74, 95% CI 1.48, 2.03). Moreover, numerous cases of the coexistence of tuberculosis and lung adenocarcinoma have been reported.

However, simultaneous occurrence of tuberculosis and carcinoma of the colon is rarely observed, and little is known about this mechanism. There are three possible explanations for this observation: (i) TB infection may be associated with the subsequent development of cancer, (ii) cancer causes reactivation of a latent TB infection, and (iii) TB and malignancy coexist.^[8]^

In general, chronic inflammatory conditions are thought to result in an impaired immune response and the development of malignancy. An increased incidence of chromosomal aberrations and excessive fibroblastic activity may lead to the development of cancer. Examples include links between gastroesophageal reflux disease and esophageal cancer, inflammatory bowel disease (ulcerative colitis and Crohn disease), and colon cancer.^[9,10]^

Reverse causality is possible because occult colon cancer may cause TB infection. Immunosuppression associated with cancer may provoke derangement of the host's intestinal mucosal barrier and reactivation of dormant TB bacilli.

In this patient, the cancer was at an advanced stage, and he had no history of TB infection. Thus, in this case, it seemed that malignancy created the proper environment for either reactivation of a latent TB infection or, more rarely, for the acquisition of a primary mycobacterial infection.

As the lesion was diagnosed as intestinal TB, clinicians must be aware of the possibility of concurrent colon adenocarcinoma and intestinal tuberculosis. Vice versa, when diagnosed with cancer, clinicians should consider the possibility of coinfection with intestinal tuberculosis, which may cause delayed anastomosis healing or postoperative infection.

## Author contributions

**Conceptualization:** Ji Hoon Kim.
